# SARS-CoV-2 Variants and Age-Dependent Infection Rates among Household and Nonhousehold Contacts

**DOI:** 10.3201/eid2908.221582

**Published:** 2023-08

**Authors:** Reiko Miyahara, Kosuke Tamura, Tomoko Kato, Mineko Nakazaki, Kanako Otani, Yura K. Ko, Taro Kamigaki, Yuzo Arima, Hideki Tani, Kazunori Oishi, Motoi Suzuki

**Affiliations:** National Institute of Infectious Diseases, Tokyo, Japan (R. Miyahara, K. Otani, T. Kamigaki, Y. Arima, M. Suzuki);; Toyama Institute of Health, Toyama, Japan (K. Tamura, M. Nakazaki, H. Tani, K. Oishi);; Takaoka Health and Welfare Center, Toyama (T. Kato);; Tohoku University Graduate School of Medicine, Miyagi, Japan (Y.K. Ko)

**Keywords:** COVID-19, 2019 novel coronavirus disease, coronavirus disease, severe acute respiratory syndrome coronavirus 2, SARS-CoV-2, viruses, respiratory infections, zoonoses, contacts, infection rates, transmission, variants of concern, household, Japan

## Abstract

To determine the effects of age and variants of concern on transmission of SARS-CoV-2, we analyzed infection rates among close contacts over 4 periods in Toyama Prefecture, Japan. Among household contacts, odds of infection were 6.2 times higher during the period of the Omicron variant than during previous periods, particularly among children and adolescents.

SARS-CoV-2 has been spreading globally since 2019; new variants of concern (VOCs) caused several epidemic waves during 2020–2022. According to a meta-analysis, the overall household secondary attack rates were higher for the Omicron variant (42.7%) than for the Alpha (36.4%) and Delta (29.7%) variants (*1*). The transmissibility and age-dependent susceptibility for Omicron and Delta exhibited significant heterogeneity among studies (*1*,*2*), and children were identified as being more vulnerable than adults to new variants (*2*). Infection rates among close contacts, determined by SARS-CoV-2 diagnostic tests, can vary according to study design, site settings, nonpharmacological control measures, and contact patterns (*3*). Thus, assessing infection rates among household and nonhousehold contacts within the same geographic area and population by using consistent methods over time could provide more reliable and valid information about changes in the effects of age and VOCs on transmission risk. With this study, we aimed to analyze the effects of age and VOCs on SARS-CoV-2 transmission by using contact tracing data of index case-patients and household and nonhousehold contacts in a city in Toyama Prefecture, Japan.

## The Study

We analyzed COVID-19 cases recorded in a city in Toyama Prefecture, Japan, over 4 periods, dominated by each of the 4 main virus variants: July 1–October 31, 2020 (pre-VOC period), April 1–30, 2021 (Alpha period), July 3–August 15, 2021 (Delta period), and January 3–23, 2022 (Omicron period) (Appendix Figure 1). Health center staff conducted telephone interviews with all COVID-19 case-patients, including those who were asymptomatic, to collect clinical information and recent activity history. According to the contact tracing guidelines of the National Institute of Infectious Diseases (Japan Ministry of Health, Labour and Welfare), we defined a close contact as someone who had contact with a COVID-19 case-patient during the period from 2 days before symptom onset until diagnosis (*4*). Close contacts were divided into household contacts (those who resided in the same household) and nonhousehold contacts (others who had contact with a confirmed COVID-19 case-patient for >15 minutes within a 1-meter distance without wearing any personal protective equipment). All contacts received SARS-CoV-2 PCR testing regardless of symptom status. If the PCR result for the first test was negative, contacts received PCR testing again if COVID-19–associated symptoms developed. We excluded from analysis close contacts with no PCR results.

All data management and analyses were conducted as part of the public health response in Toyama Prefecture and the National Institute of Infectious Diseases, and we used registered data collected according to the Infectious Diseases Law of Japan. Ethics approval was not required for this study.

First, we determined the baseline characteristics of the index case-patients and close contacts for each of the 4 periods. Second, we calculated infection rates stratified by the characteristics of index case-patients (age, sex, history of contact with COVID-19 case-patients before diagnosis, and symptom status) and close contacts (age, sex, and interval between diagnosis of index case-patients and PCR results of contacts). To adjust for clustering effects, we calculated infection rates as the total number of positive contacts divided by the total number of close contacts (with 95% CIs) by using the svyset command in Stata (StataCorp LLC, https://www.stata.com). To account for clustering among contacts exposed to the same index case-patients, we analyzed odds ratios of the infection rates (with 95% CIs) by using GEE (generalized estimating equations) logistic regression models with exchangeable correlations. We adjusted the models for the characteristics of both the index case-patients and their close contacts. Third, we described the contact matrix for the average number of contacts and infection rates based on the age of the index case-patients and contacts. We used Stata version 16.0 and R version 4.2.1 (The R Project for Statistical Computing, https://www.r-project.org) software to perform statistical analyses. 

We enrolled 1,057 patients and 3,820 contacts: 123 index case-patients and 530 close contacts, in the pre-VOC period; 246 index case-patients and 988 close contacts in the Alpha period; 304 index case-patients and 984 close contacts in the Delta period; and 384 index case-patients and 1,318 close contacts in the Omicron period (Appendix Table 1). We excluded close contacts without PCR results: 45 (8.5%) persons from the pre-VOC period, 29 (2.9%) from the Alpha period, 111 (11.3%) from the Delta period, and 173 (13.1%) from the Omicron period. Infection rates during the Omicron period were 35.0% (95% CI 28.3–42.2) for household contacts and 15.1% (95% CI 10.0–22.5) for nonhousehold contacts. After adjustment for age, symptoms, sex, contact history, interval from diagnosis of index case-patient to PCR test, and household size, the odds ratios for infection were 6.22 times higher among household contacts and 3.55 times higher among nonhousehold contacts during the Omicron period than during the pre-VOC period (Table; Appendix Table 2). The risk for infection among household contacts 0–19 years of age increased significantly, from 3% in the pre-VOC period to 38% during the Omicron period (Appendix Figure 2). In contrast, during the study period, infection rates for nonhousehold contacts in this age group were lower, despite a higher number of contacts compared with nonhousehold contacts in other age groups (Appendix Figure 3). Infection rates among household contacts >60 years of age decreased during the Delta period (12%) but increased again during the Omicron period (29%). Regarding infectivity throughout all time periods, the risk for infection from index case-patients >60 years of age was higher than that from index case-patients of other ages (Appendix Figure 2). 

**Table:** Infection rates of SARS-CoV-2 infection among household and nonhousehold contacts in study of SARS-CoV-2 variants and age-dependent infection rates

Variable	Household contacts					Nonhousehold contacts			
	Total no.	No. PCR positive	Infection rate, %	Adjusted odds ratio (95% CI)*		Total no.	No. PCR positive	Infection rate, %	Adjusted odds ratio (95% CI)*
Total	1,144	294	25.7			2,318	302	13.0	
Period									
Pre-VOC	155	20	12.9	Referent		330	36	10.9	Referent
Alpha	251	48	19.1	1.91 (0.94–3.90)		708	71	10.0	1.47 (0.86–2.50)
Delta	329	83	25.2	3.75 (1.84–7.61)		544	84	15.4	2.34 (1.37–3.98)
Omicron	409	143	35.0	6.22 (3.04–12.70)		736	111	15.1	3.55 (2.09–6.06)
Index case-patient age, y									
0–19	214	54	25.2	0.42 (0.20–0.86)		852	34	4.0	0.16 (0.08–0.34)
20–39	493	111	22.5	0.36 (0.20–0.66)		973	182	18.7	0.42 (0.25–0.73)
40–59	309	84	27.2	0.45 (0.24–0.83)		317	48	15.1	0.40 (0.22–0.72)
>60	129	45	35.2	Referent		176	38	21.6	Referent
Close contact age, y									
0–19	295	80	27.1	1.06 (0.70–1.62)		831	45	5.4	0.67 (0.39–1.17)
20–39	259	79	30.5	1.33 (0.89–2.00)		721	162	22.5	1.09 (0.70–1.71)
40–59	359	84	23.4	1.14 (0.78–1.68)		353	38	10.8	0.52 (0.31–0.85)
>60	227	51	22.5	Referent		257	51	19.8	Referent
Unknown	4	0	0	NA		156	6	3.8	0.22 (0.07–0.66)

* Odds ratios were adjusted for age, sex, symptoms of index case-patients at the time of diagnosis, contact history, interval from diagnosis of index case-patient to PCR tests, and number of persons in the same household. NA, not applicable; VOC, variant of concern.

## Conclusions

Our study showed that odds of infection were 6.2 times higher for household contacts during the Omicron period than during the pre-VOC period and that children and adolescents were particularly vulnerable (*2*). Despite increased nonhousehold contact among persons 0–19 years of age, nonphysical contact (*5*) and nonpharmacological control measures (*6*) in school and daycare centers may have led to lower infection rates and fewer large outbreaks in schools.

In addition, infection rates for contacts >60 years of age decreased during the Delta period but increased again during the Omicron period, potentially because of waning immunity associated with SARS-CoV-2 vaccination and the attenuated effect on the Omicron variant (*7*), even with high vaccination rates (93%) among persons >65 years of age during the Omicron period (Appendix Figure 1). In addition, the infectivity of elderly persons tended to be higher than that of persons in other age groups even after vaccine introduction (*8*), possibly because of close contact, such as caregiving and nursing care. The value of protecting those who care for elderly case-patients should thus be emphasized.

A limitation of this study was the varied timing and frequency of PCR testing. As the number of days from symptom onset to diagnosis decreased over time, infection rates were associated with the timing of testing and symptoms at the time of testing. We might have missed asymptomatic infections and potentially overcounted infected case-patients among contacts who might have been exposed to other places or infected persons.

Our finding of increased odds of infection among household contacts during the period of the Omicron variant, particularly among children and adolescents, highlights the need for periodic surveys to investigate comparative infectivity by epidemic strain as well as susceptibility and trends by age group over time in the same area and population. Such studies would account for variations in local conditions such as control regulation, contract tracing strategy, population age structure, and vaccination coverage.

Appendix Additional results from study of SARS-CoV-2 variants and age-dependent infection rates.
