# Supplementary material for: SARS-CoV-2 Variants and Age-Dependent Infection Rates among Household and Nonhousehold Contacts
Source: Emerg Infect Dis. 2023 Aug;29(8):1648–50. doi: 10.3201/eid2908.221582 (PMC10370865; doi:10.3201/eid2908.221582)
Supplement: Appendix — Additional results from study of SARS-CoV-2 variants and age-dependent infection rates. [file 22-1582-Techapp-s1.pdf]

Article DOI: https://doi.org/10.3201/eid2908.221582

EID cannot ensure accessibility for supplementary materials supplied by authors. Readers who have difficulty accessing supplementary content should contact the authors for assistance.

SARS-CoV-2 Variants and Age-Dependent Infection Rates among Household and Nonhousehold Contacts

Appendix

Appendix Table 1. Characteristics of index patients and contacts in the pre-VOC period, the Alpha period, the Delta period and the Omicron period

Category	Subcategory	pre-VOC period July 2020 – October 2020	Alpha period April 2021	Delta period July-August 2021	Omicron period January 2022
Index case-patients		n = 123	n = 246	n = 304	n = 384
Age (years), n (%)	0–19	4 (3.3)	29 (11.8)	32 (10.5)	81 (21.1)
	20–39	56 (45.5)	95 (38.6)	171 (56.3)	184 (47.9)
	40–59	26 (21.1)	65 (26.4)	83 (27.3)	78 (20.3)
	≥60	37 (30.1)	57 (23.2)	18 (5.9)	41 (10.7)
Sex, n (%)	Male	70 (56.9)	153 (62.2)	175 (57.6)	209 (54.4)
	Female	53 (43.1)	93 (37.8)	129 (42.4)	175 (45.6)
Variants of concern, n (%)	Non-VOC	123 (100)	47 (19.1)		
	Alpha		145 (58.9)	31 (10.2)	
	Delta			273 (89.8)	13 (3.4)
	Omicron				68 (17.7)
	Unclassified / untested case-patients		54 (7.3)		303 (78.9)
Symptomatic at time of diagnosis, n (%)	Yes	104 (84.6)	202 (82.1)	294 (96.7)	351 (91.4)
Contact history, n(%)	Yes	77 (62.6)	174 (70.7)	186 (61.2)	184 (47.9)
Days from symptom onset to diagnosis	Mean days (SD)	5.31 (4.67)	3.82 (3.17)	2.80 (2.09)	2.13 (2.27)
Index case who had close contacts,, n(%)	Yes	99 (80.5)	161 (65.4)	195 (64.1)	253 (65.9)
Number of close contacts	Mean (median)	5.35 (3)	6.14 (3)	5.05 (3)	5.21 (3)
Number of familial contacts	Mean (median)	1.58 (1)	1.57 (1)	1.69 (2)	1.73 (2)
Contacts		n = 530	n = 988	n = 984	n = 1,318
Age (years), n (%)	0–19	61 (11.5)	383 (38.8)	219 (22.3)	478 (36.3)

Category	Subcategory	pre-VOC period<br>July 2020 – October 2020	Alpha period<br>April 2021	Delta period<br>July-August 2021	Omicron period<br>January 2022
	20–39	166 (31.3)	252 (25.5)	326 (33.1)	292 (22.2)
	40–59	130 (24.5)	173 (17.5)	235 (23.9)	206 (15.6)
	≥60	95 (17.9)	132 (13.4)	98 (10.0)	175 (13.3)
	Unknown	78 (14.7)	48 (4.9)	106 (10.8)	167 (12.7)
Sex, n (%)	Male	269 (50.8)	542 (54.9)	490 (49.8)	585 (44.4)
	Female	217 (10.9)	442 (44.7)	428 (43.5)	603 (45.8)
	Unknown	44 (8.3)	4 (0.4)	66 (6.7)	130 (9.9)
Symptomatic at time of PCR test, n (%)	Yes	94 (17.7)	250 (25.3)	181 (18.4)	261 (19.8)
PCR test result, n (%)	Negative	429 (80.9)	840 (85.0)	706 (71.8)	891 (67.6)
	Positive	56 (10.6)	119 (12.0)	167 (17.0)	254 (19.3)
	Untested/Unknown	45 (8.5)	29 (2.9)	111 (11.3)	173 (13.1)
Type of contact, n (%)	Household	156 (29.4)	253 (25.6)	332 (33.7)	437 (33.2)
	Non-household	375 (70.6)	735 (74.4)	652 (66.3)	881 (66.8)
Place of contact, n (%)	Home	235 (44.3)	357 (36.1)	444 (45.1)	566 (42.9)
	School	0 (0)	141 (14.3)	93 (9.5)	194 (14.7)
	Hospital/Nursing home	46 (8.7)	22 (2.2)	19 (1.9)	80 (6.1)
	Nursery	0 (0)	197 (19.9)	45 (4.6)	135 (10.2)
	Restaurants/eating	110 (20.8)	93 (9.4)	140 (14.2)	168 (12.8)
	Work place	87 (16.4)	93 (9.4)	171 (17.4)	66 (5.0)
	Outside	6 (1.1)	30 (3)	24 (2.4)	35 (2.7)
	Inside	10 (1.9)	23 (2.3)	25 (2.5)	8 (0.6)
	Others	36 (6.8)	32 (3.2)	23 (2.3)	66 (5)
Interval from diagnosis of index case to PCR test, n (%)	0–1day	276 (52.1)	286 (29.0)	185 (18.8)	210 (15.9)
	2–3 d	163 (30.8)	412 (41.7)	557 (56.6)	543 (41.2)
	4–5 d	9 (1.7)	26 (2.6)	63 (6.4)	298 (22.6)
	6–10 d	9 (1.7)	27 (2.7)	59 (6.0)	58 (4.4)
	≥11 d	1 (0.2)	29 (2.9)	8 (0.8)	9 (0.7)
	Untested/Unknown	72 (13.6)	208 (21.1)	112 (11.4)	200 (15.2)
Symptomatic case-patients among PCR positive contacts		38/56 (67.9%)	91/119 (76.5%)	145/167 (86.8%)	204/254 (80.3%)

Appendix Table 2. Infection rates of SARS-CoV-2 infection among household and non-household contacts

Category	Sub-category	Household contacts Number	PCR positive	Infection rates(%)	Odds ratio (95% CI)	Adjusted odds ratio (95% CI)	Non-household contacts Number	PCR positive	Infection rates (%)	Odds ratio (95% CI)	Adjusted odds ratio (95% CI)
		N=1,144	N=294	25.7			N=2,318	N=302	13.0		
Index case characteristics											
Time period	pre-VOC	155	20	12.9	ref	ref	330	36	10.9	ref	ref
	Alpha	251	48	19.1	1.41 (0.73, 2.74)	1.91 (0.94, 3.90)	708	71	10.0	1.15 (0.61,2.17)	1.47 (0.86,2.50)
	Delta	329	83	25.2	1.91 (1.02, 3.58)	3.75 (1.84,7.61)	544	84	15.4	1.67 (0.92,3.06)	2.34 (1.37,3.98)
	Omicron	409	143	35.0	2.77 (1.51, 5.05)	6.22 (3.04,12.70)	736	111	15.1	2.18 (1.22, 3.92)	3.55 (2.09,6.06)
Symptomatic at the time of diagnosis	No	72	13	18.1	ref	ref	207	5	2.4	ref	ref
	Yes	1,073	281	26.2	1.77 (0.81, 3.90)	1.59 (0.66,3.83)	2,111	297	14.1	2.20 (0.73, 6.67)	2.32 (0.78,6.89)
Age (years)	0-19	214	54	25.2	0.55 (0.29,1.04)	0.42 (0.20,0.86)	852	34	4.0	0.23 (0.11,0.46)	0.16 (0.08,0.34)
	20-39	493	111	22.5	0.54 (0.32,0.92)	0.36 (0.20,0.66)	973	182	18.7	0.73 (0.45,1.16)	0.42 (0.25,0.73)
	40-59	309	84	27.2	0.64 (0.37,1.11)	0.45 (0.24,0.83)	317	48	15.1	0.71 (0.41,1.24)	0.40 (0.22,0.72)
	≥60	129	45	35.2	ref	ref	176	38	21.6	ref	ref
Sex	Male	713	186	26.1	ref	ref	1,419	187	13.2	ref	ref
	Female	432	108	25.0	0.91 (0.64,1.30)	0.98 (0.67,1.42)	899	115	12.8	0.85 (0.59, 1.23)	0.84 (0.60,1.18)
Contact history	No	620	196	31.6	ref	ref	1,431	214	15.0	ref	ref
	Yes	525	98	18.7	0.56 (0.39,0.79)	0.67 (0.46,0.99)	887	88	9.9	0.60 (0.41,0.88)	0.70 (0.49,0.99)
Close contact characteristics											
Age (years)	0-19	295	80	27.1	0.86 (0.61,1.22)	1.06 (0.70,1.62)	831	45	5.4	0.45 (0.28,0.72)	0.67 (0.39,1.17)
	20-39	259	79	30.5	1.23 (0.87,1.73)	1.33 (0.89,2.00)	721	162	22.5	0.96 (0.66,1.40)	1.09 (0.70,1.71)
	40-59	359	84	23.4	0.99 (0.72,1.37)	1.14 (0.78,1.68)	353	38	10.8	0.52 (0.33,0.80)	0.52 (0.31,0.85)
	≥60	227	51	22.5	ref	ref	257	51	19.8	ref	ref
	Unknown	4	0	0	-	-	156	6	3.8	0.10 (0.03,0.30)	0.22 (0.07,0.66)
Sex	Male	492	119	24.2	ref	ref	1,287	179	13.9	ref	ref
	female	646	175	27.1	1.17 (0.94,1.45)	1.18 (0.91,1.51)	954	121	12.7	0.97 (0.78,1.20)	1.00 (0.76,1.33)
	Unknown	7	0	0	-	-	77	2	2.6	0.06 (0.01,0.57)	0.25 (0.04,1.85)
Interval from diagnosis of index case to PCR test	0-1day	387	111	28.7	ref	ref	567	129	22.8	Ref	ref
	2-3 days	501	102	20.4	0.53 (0.37,0.76)	0.37 (0.24,0.56)	1,174	116	9.9	0.33 (0.24,0.45)	0.28 (0.20,0.38)
	4-5 days	170	35	20.6	0.55 (0.34,0.90)	0.29 (0.17,0.50)	226	27	11.9	0.45 (0.27,0.75)	0.34 (0.20,0.59)
	6-10 days	57	30	52.6	1.17 (0.67,2.05)	0.91 (0.49,1.68)	96	16	16.7	0.81 (0.42,1.57)	0.63 (0.31,1.26)
	.≥11 days	28	15	53.6	0.89 (0.42,1.92)	0.78 (0.34,1.78)	19	9	47.4	1.04 (0.37,2.95)	1.34 (0.46,3.88)
	Unknown	1	1	100.0	-	-	236	5	2.1	0.09 (0.02,0.37)	0.46 (0.15,1.40)

Category	Sub-category	Household contacts Number	PCR positive	Infection rates(%)	Odds ratio (95% CI)	Adjusted odds ratio (95% CI)	Non-household contacts Number	PCR positive	Infection rates (%)	Odds ratio (95% CI)	Adjusted odds ratio (95% CI)
		N=1,144	N=294	25.7			N=2,318	N=302	13.0		
Number of people in the same household	1-2	427	126	29.5	ref	ref					
	3-5	625	150	24.0	0.70 (0.49,1.00)	0.74 (0.51,1.08)					
	over 6	92	18	19.6	0.57 (0.20,1.62)	0.52 (0.19,1.43)					

Appendix Table 3a. Infection rates of SARS-CoV-2 infection among household and non-household contacts, pre-VOC period

Category	Sub-category	Household contacts Number	PCR positive	Infection rate (%)	Odds Ratio (95% CI)	Adjusted Odds ratio (95% CI)	Non-household contacts Number	PCR positive	Infection rate (%)	Odds Ratio (95% CI)	Adjusted Odds ratio (95% CI)
Total		N=155	N=20	12.9			N=330	N=36	10.9		
Index case characteristics											
Age (years)	0-19	32	0	0	-	-	1	0	0	-	-
	20-39	75	5	6.7	0.25 (0.08,0.75)	0.24 (0.05,1.12)	171	19	11.1	0.41 (0.16,1.03)	0.16 (0.04,0.72)
	40-59	28	4	14.3	0.57 (0.16,2.00)	0.55 (0.11,2.83)	97	3	3.1	0.22 (0.05,0.88)	0.08 (0.01,0.53)
	≥60	49	11	22.4	ref	ref	61	14	23.0	ref	ref
Sex	Male	94	13	13.8	ref	ref	204	23	11.3	ref	ref
	Female	61	7	11.5	0.79 (0.29,2.15)	0.49 (0.10,2.53)	126	13	10.3	1.28 (0.5,3.29)	0.68 (0.23,1.99)
Symptomatic	No	20	3	15.0	ref	ref	17	1	5.9	ref	ref
	Yes	135	17	12.6	0.82 (0.21,3.12)	2.04 (0.25,16.82)	313	35	11.2	1.78 (0.22,14.44)	1.92 (0.17,22.25)
Contact history	No	70	10	14.3	ref	ref	196	26	13.3	ref	ref
	Yes	85	10	11.8	0.81 (0.31,2.09)	0.79 (0.23,2.72)	134	10	7.5	0.42 (0.16,1.14)	0.19 (0.06,0.60)
Close contact characteristics											
Age	0-19	34	1	2.9	0.16 (0.02,1.35)	0.38 (0.04,3.97)	27	0	0	-	-
	20-39	33	8	24.2	1.66 (0.54,5.1)	4.02 (0.94,17.2)	120	19	15.8	0.64 (0.28,1.49)	1.21 (0.34,4.27)
	40-59	40	4	10.0	0.60 (0.16,2.15)	0.73 (0.14,3.80)	87	3	3.4	0.23 (0.08,0.67)	0.29 (0.07,1.09)
	≥60	44	7	15.9	ref	ref	48	14	29.2	ref	ref

Category	Sub-category	Household contacts Number	PCR positive	Infection rate (%)	Odds Ratio (95% CI)	Adjusted Odds ratio (95% CI)	Non-household contacts Number	PCR positive	Infection rate (%)	Odds Ratio (95% CI)	Adjusted Odds ratio (95% CI)
	Unknown	4	0	0	-	-	48	0	0	-	-
Sex	Male	67	9	13.4	ref	ref	178	18	10.1	ref	ref
	female	87	11	12.6	0.92 (0.36,2.35)	1.12 (0.28,4.39)	124	18	14.5	1.07 (0.57,2.03)	0.98 (0.44,2.19)
	Unknown	1	0	0	-	-	28	0	0	-	-
Interval from diagnosis of index case to PCR test	0-1day	124	14	11.3	ref	ref	152	25	16.4	ref	ref
	2-3 days	26	2	7.7	0.66 (0.14,3.08)	0.37 (0.06,2.17)	137	10	7.3	0.67 (0.28,1.58)	0.72 (0.25,2.07)
	4-5 days	3	2	66.7	15.7 (1.34,184.62)	4.34 (0.24,79.14)	6	1	16.7	0.55 (0.04,7.08)	0.90 (0.07,11.88)
	6-10 days	1	1	100	-	-	9	0	0	-	-
	≥11 days	1	1	100	-	-	26	0	0	-	-
Number of same household	1-2	62	13	21.0	ref	ref					
	3-5	85	5	5.9	0.23 (0.08,0.67)	0.39 (0.10,1.45)					
	over 6	8	2	25.0	1.28 (0.29,5.63)	0.74 (0.07,8.02)					

Appendix Table 3b. Infection rates of SARS-CoV-2 infection among household and non-household contacts, Alpha period

Category	Sub-category	Household contacts Number	PCR positive	Infection rate (%)	Odds Ratio (95% CI)	Adjusted Odds ratio (95% CI)	Non-household contacts Number	PCR positive	Infection rate (%)	Odds Ratio (95% CI)	Adjusted Odds ratio (95% CI)
Total		251	48	19.1			708	71	10		
Index case characteristics											
Age	0-19	58	6	10.3	0.16 (0.04,0.61)	0.26 (0.05,1.30)	329	9	2.7	0.29 (0.07,1.19)	0.20 (0.04,1.07)
	20-39	77	8	10.4	0.20 (0.06,0.59)	0.17 (0.05,0.64)	227	38	16.7	0.77 (0.31,1.89)	0.32 (0.10,1.03)
	40-59	80	20	25.0	0.55 (0.22,1.39)	0.44 (0.14,1.39)	70	13	18.6	1.20 (0.44,3.27)	0.78 (0.25,2.46)
	≥60	36	14	38.9	ref	ref	82	11	13.4	ref	ref
Sex	Male	159	26	16.4	ref	ref	524	55	10.5	ref	ref
	Female	92	22	23.9	1.42 (0.67,2.99)	1.46 (0.62,3.45)	184	16	8.7	0.68 (0.29,1.58)	0.51 (0.22,1.17)
Symptomatic	No	22	2	9.1	ref	ref	182	3	1.6	ref	ref

Category	Sub-category	Household contacts Number	PCR positive	Infection rate (%)	Odds Ratio (95% CI)	Adjusted Odds ratio (95% CI)	Non-household contacts Number	PCR positive	Infection rate (%)	Odds Ratio (95% CI)	Adjusted Odds ratio (95% CI)
	Yes	229	46	20.1	2.73 (0.49,15.19)	2.28 (0.40,13.10)	526	68	12.9	1.98 (0.39,10.12)	2.72 (0.48,15.41)
Contact history	No	100	29	29.0	ref	ref	275	34	12.4	ref	ref
	Yes	151	19	12.6	0.41 (0.20,0.86)	0.76 (0.33,1.74)	433	37	8.5	0.85 (0.39,1.83)	1.26 (0.61,2.61)
Close contact characteristics											
Age	0-19	59	8	13.6	0.42 (0.17,1.05)	0.99 (0.29,3.35)	321	13	4.0	0.98 (0.34,2.83)	2.63 (0.65,10.73)
	20-39	65	9	13.8	0.47 (0.19,1.15)	0.82 (0.26,2.59)	175	37	21.1	1.15 (0.48,2.77)	1.88 (0.64,5.51)
	40-59	72	16	22.2	0.82 (0.39,1.75)	1.51 (0.52,4.38)	97	9	9.3	0.74 (0.29,1.91)	0.92 (0.32,2.70)
	≥60	55	15	27.3	ref	ref	76	12	15.8	ref	ref
	Unknown	0	0	0	-	-	39	0	0	-	-
Sex	Male	112	18	16.1	ref	ref	412	53	12.9	ref	ref
	female	139	30	21.6	1.28 (0.71,2.33)	1.21 (0.56,2.63)	293	18	6.1	0.81 (0.53,1.24)	0.78 (0.40,1.52)
	Unknown	0	0	0	-	-	3	0	0	-	-
Interval from diagnosis of index case to PCR test	0-1day	112	22	19.6	ref	ref	171	29	17.0	ref	ref
	2-3 days	104	11	10.6	0.47 (0.20,1.11)	0.47 (0.18,1.20)	308	17	5.5	0.22 (0.11,0.47)	0.20 (0.09,0.42)
	4-5 days	9	5	55.6	5.08 (1.12,22.93)	8.08 (1.30,50.14)	17	7	41.2	1.75 (0.58,5.28)	1.73 (0.53,5.65)
	6-10 days	13	5	38.5	2.12 (0.58,7.67)	1.73 (0.40,7.41)	14	9	64.3	4.76 (1.55,14.6)	4.27 (1.26,14.51)
	≥11 days	13	5	38.5	1.55 (0.42,5.78)	1.90 (0.47,7.65)	16	9	56.3	1.86 (0.59,5.89)	2.36 (0.73,7.69)
Number of same household	1-2	108	26	24.1	ref	ref					
	3-5	112	20	17.9	0.66 (0.31,1.41)	1.05 (0.42,2.59)					
	over 6	31	2	6.5	0.23 (0.02,2.29)	0.48 (0.06,3.68)					

Appendix Table 3c. Infection rates of SARS-CoV-2 infection among household and non-household contacts, Delta period

		Household contacts					Non-household contacts				
Category	Sub-category	Number	PCR positive	Infection rate (%)	Odds Ratio (95% CI)	Adjusted Odds ratio (95% CI)	Number	PCR positive	Infection rate (%)	Odds Ratio (95% CI)	Adjusted Odds ratio (95% CI)
Total		329	83	25.2			544	84	15.4		
Index case characteristics											
Age	0-19	31	9	29.0	0.74 (0.14,3.86)	0.85 (0.11,6.33)	134	7	5.2	0.29 (0.05,1.76)	1.50 (0.20,11.45)
	20-39	183	42	23.0	0.56 (0.14,2.21)	0.41 (0.08,2.22)	283	57	20.1	0.99 (0.22,4.49)	1.31 (0.25,6.81)
	40-59	93	23	24.7	0.53 (0.13,2.26)	0.39 (0.07,2.22)	111	16	14.4	0.55 (0.11,2.78)	0.72 (0.13,3.92)
	≥60	22	9	40.9	ref	ref	16	4	25.0	ref	ref
Sex	Male	208	50	24.0	ref	ref	345	51	14.8	ref	ref
	Female	121	33	27.3	1.22 (0.64,2.35)	1.40 (0.65,2.99)	199	33	16.6	0.80 (0.41,1.56)	1.14 (0.56,2.32)
Symptomatic	No	4	1	25.0	ref	ref	3	0	0	-	-
	Yes	325	82	25.2	0.60 (0.05,7.47)	0.54 (0.02,14.7)	541	84	15.5	-	-
Contact history	No	171	47	27.5	ref	ref	306	61	19.9	ref	ref
	Yes	158	36	22.8	0.82 (0.43,1.55)	0.70 (0.33,1.47)	238	23	9.7	0.50 (0.26,0.98)	0.38 (0.19,0.75)
Close contact characteristics											
Age	0-19	76	23	30.3	1.21 (0.57,2.56)	1.39 (0.60,3.22)	142	5	3.5	0.41 (0.10,1.59)	0.34 (0.08,1.50)
	20-39	72	27	37.5	1.87 (0.93,3.77)	1.53 (0.70,3.31)	29	57	23.8	2.39 (0.89,6.38)	1.91 (0.66,5.54)
	40-59	131	27	20.6	1.09 (0.55,2.17)	1.15 (0.53,2.46)	100	15	15.0	1.46 (0.51,4.21)	1.21 (0.40,3.63)
	≥60	50	6	12.0	ref	ref	44	5	11.4	ref	ref
	Unknown	0	0	0	-	-	19	2	10.5	1.22 (0.24,6.18)	0.56 (0.07,4.81)
Sex	Male	140	34	24.3	ref	ref	327	49	15.0	ref	ref
	female	189	49	25.9	1.14 (0.76,1.70)	1.23 (0.78,1.95)	210	34	16.2	0.95 (0.57,1.57)	1.14 (0.61,2.11)
	Unknown	0	0	0	-	-	7	1	14.3	1.20 (0.17,8.35)	-
Interval from diagnosis of index case to PCR test	0-1day	83	34	41.0	ref	ref	102	31	30.4	ref	ref
	2-3 days	198	33	16.7	0.24 (0.12,0.47)	0.21 (0.10,0.45)	359	44	12.3	0.22 (0.12,0.39)	0.19 (0.10,0.36)
	4-5 days	24	4	16.7	0.19 (0.05,0.74)	0.13 (0.03,0.55)	39	7	17.9	0.31 (0.11,0.88)	0.28 (0.09,0.82)
	6-10 days	19	9	47.4	0.55 (0.19,1.57)	0.40 (0.13,1.24)	40	1	2.5	0.24 (0.04,1.59)	0.40 (0.04,4.04)
	≥11 days	5	3	60.0	0.53 (0.10,2.92)	0.41 (0.07,2.51)	3	0	0	-	-
Number of same household	1-2	118	40	33.9	ref	ref					
	3-5	187	38	20.3	0.49 (0.25,0.96)	0.39 (0.18,0.83)					
	over 6	24	5	20.8	0.49 (0.08,3.14)	0.32 (0.03,3.62)					

Appendix Table 3d. Infection rates of SARS-CoV-2 infection among household and non-household contacts, Omicron period

		Household contacts					Non-household contacts				
Category	Sub-category	Number	PCR positive	Infection rate (%)	Odds Ratio (95% CI)	Adjusted Odds ratio (95% CI)	Number	PCR positive	Infection rate (%)	Odds Ratio (95% CI)	Adjusted Odds ratio (95% CI)
Total		409	143	35.0			736	111	15.1		
Index case characteristics											
Age	0-19	122	39	32.0	0.39 (0.13,1.24)	0.44 (0.12,1.62)	388	18	4.6	0.06 (0.02,0.2)	0.04 (0.01,0.22)
	20-39	158	56	35.4	0.51 (0.17,1.51)	0.51 (0.15,1.67)	292	68	23.3	0.25 (0.09,0.72)	0.12 (0.03,0.49)
	40-59	108	37	34.3	0.45 (0.14,1.40)	0.51 (0.15,1.75)	39	16	41.0	0.60 (0.18,2.00)	0.29 (0.06,1.39)
	≥60	21	11	52.4	ref	ref	17	9	52.9	ref	ref
Sex	Male	251	97	38.6	ref	ref	346	58	16.8	ref	ref
	Female	158	46	29.1	0.62 (0.35,1.12)	0.70 (0.37,1.33)	390	53	13.6	0.81 (0.43,1.52)	1.10 (0.57,2.13)
Symptomatic	No	26	7	26.9	ref	ref	5	1	20.0	ref	ref
	Yes	383	136	35.5	1.87 (0.50,6.90)	1.40 (0.32,6.07)	731	110	15.0	0.93 (0.09,9.31)	5.16 (0.45,59.14)
Contact	No	279	110	39.4	ref	ref	654	93	14.2	ref	ref
history	Yes	130	33	25.4	0.50 (0.27,0.95)	0.52 (0.25,1.07)	82	18	22	0.96 (0.46,1.99)	0.69 (0.31,1.53)
Close contact characteristics											
Age	0-19	126	48	38.1	1.03 (0.62,1.70)	1.07 (0.60,1.91)	341	27	7.9	0.53 (0.29,0.98)	1.19 (0.47,3.01)
	20-39	89	35	39.3	1.28 (0.75,2.16)	1.11 (0.61,2.01)	187	49	26.2	0.55 (0.30,1.00)	1.41 (0.58,3.43)
	40-59	116	37	31.9	1.14 (0.70,1.85)	1.17 (0.67,2.04)	69	11	15.9	0.27 (0.13,0.57)	0.60 (0.21,1.69)
	≥60	78	23	29.5	ref	ref	89	20	22.5	ref	ref
	Unknown	0	0	0	-	-	50	4	8.0	0.10 (0.03,0.36)	0.68 (0.14,3.36)
Sex	Male	173	58	33.5	ref	ref	370	59	15.9	ref	ref
	female	230	85	37.0	1.24 (0.92,1.67)	1.28 (0.90,1.82)	327	51	15.6	0.96 (0.72,1.29)	0.93 (0.59,1.45)
	Unknown	6	0	0	-	-	39	1	2.6	-	-
Interval from	0-1day	66	41	60.3	ref	ref	142	44	31.0	ref	ref
diagnosis of	2-3 days	173	56	32.4	0.30 (0.16,0.58)	0.31 (0.15,0.60)	370	45	12.2	0.22 (0.13,0.40)	0.22 (0.12,0.4)0
index case to	4-5 days	134	24	17.9	0.18 (0.09,0.36)	0.17 (0.08,0.34)	164	12	7.3	0.12 (0.05,0.30)	0.12 (0.05,0.32)
PCR test	6-10 days	25	16	64.0	0.50 (0.21,1.18)	0.51 (0.20,1.27)	33	6	18.2	0.22 (0.06,0.80)	0.21 (0.06,0.76)
	≥11 days	11	6	66.7	0.44 (0.13,1.45)	0.4 0(0.11,1.38)	0	0	0	-	-
Number of	1-2	139	47	33.8	ref	ref					
same	3-5	241	87	36.1	0.97 (0.55,1.71)	1.28 (0.68,2.40)					
household	over 6	29	9	31.0	0.88 (0.16,4.87)	1.06 (0.18,6.22)					

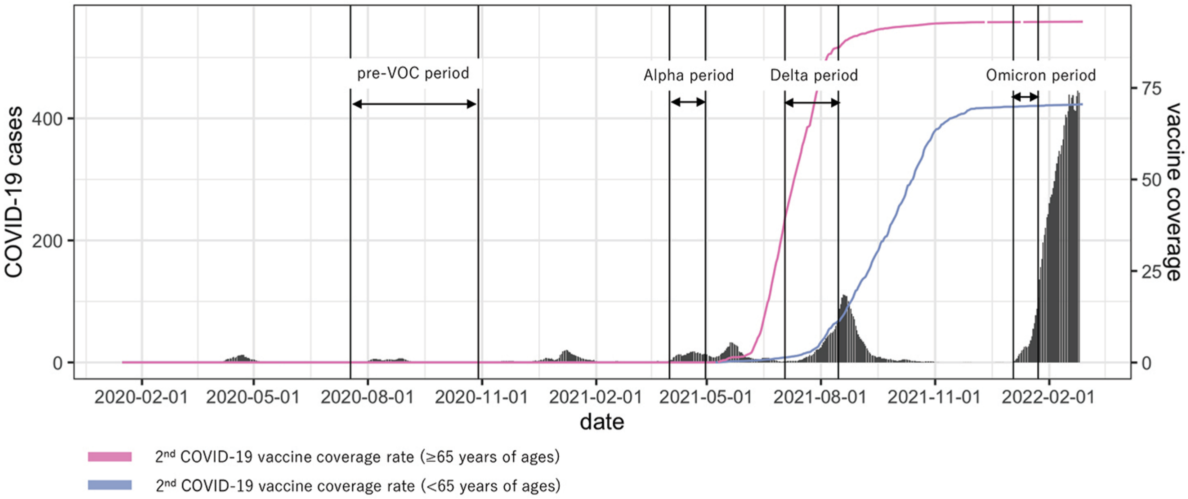

Appendix Figure 1. COVID-19 case-patients and vaccination coverage rates in Toyama Prefecture

Vaccination coverage was defined as the percentage of people in the population who received the 2nd dose of COVID-19 vaccine. The number of people receiving the 2nd dose of COVID-19 vaccines in Toyama prefecture were reported in the Web site of the Digital Agency, Japan (https://info.vrs.digital.go.jp/opendata). The denominator used for vaccine coverage was the total number of population as of October 1, 2020, published in the Web site of e-Stat https://www.e-stat.go.jp. COVID-19 case-patients were reported from the Toyama prefecture. We downloaded the database from NHK, openly available at https://www3.nhk.or.jp/news/special/coronavirus/data.

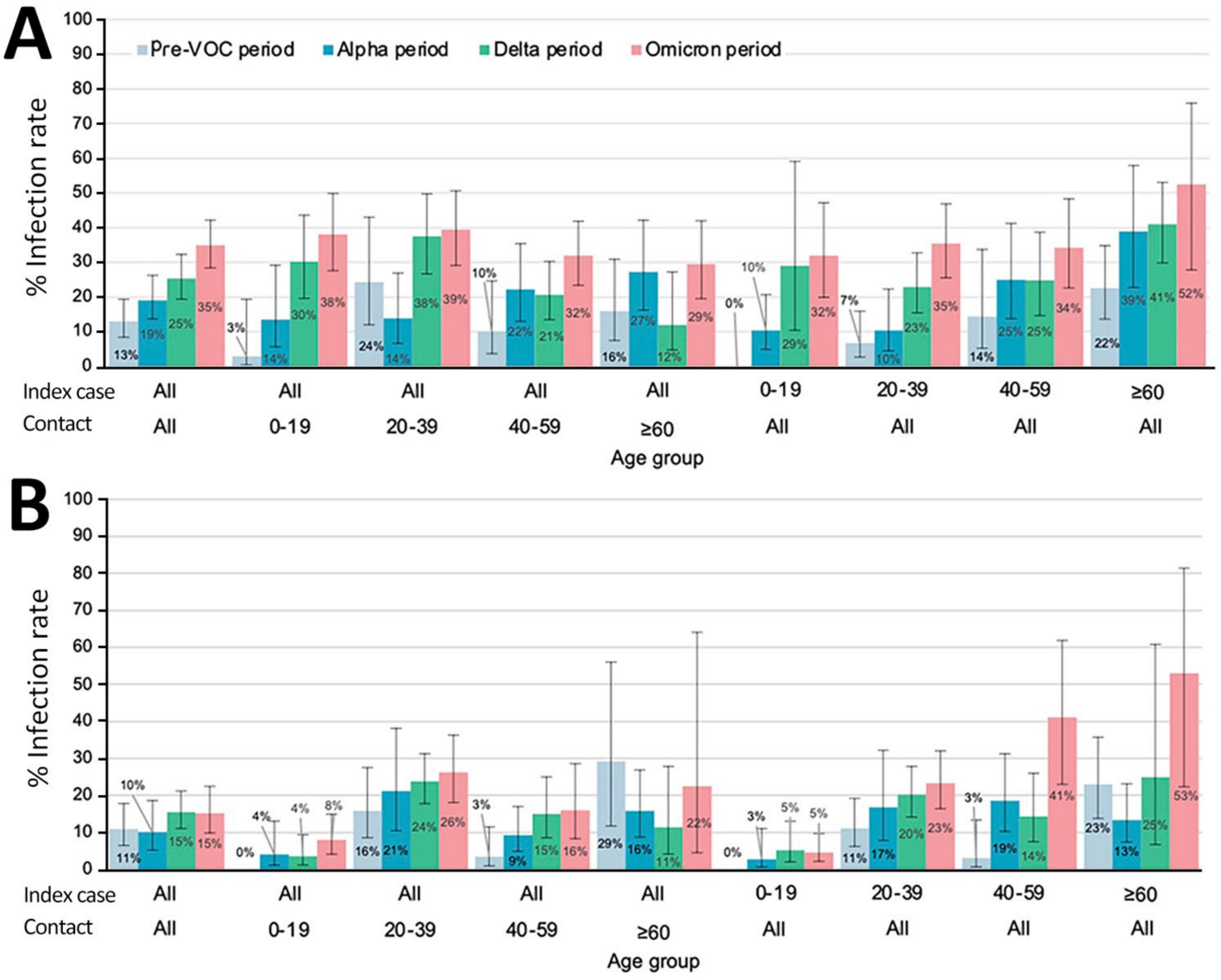

Appendix Figure 2. Age -specific infection rates according to age of the index case-patient and contact stratified by household contacts (A) and nonhousehold contacts (B) in study of SARS-CoV-2 variants and age-dependent infection rates. Age groups were categorized as 0–19, 20–39, 40–59, and ≥60 years. “All” includes all age groups. The upper row of the age group shows the age of index case-patients, and the lower row shows the age of contacts. Error bars indicate 95% CIs.

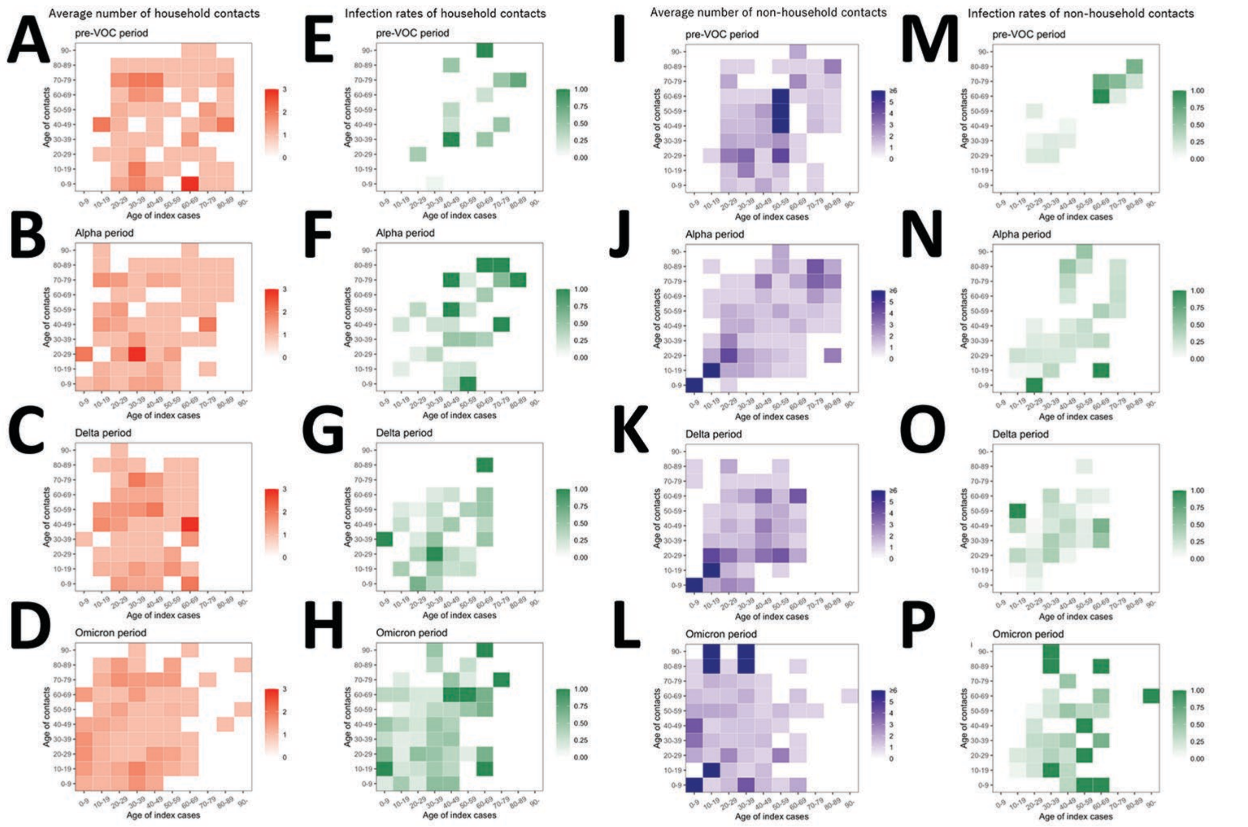

Appendix Figure 3. Age-structured matrices for the average number of contacts and infection rates of SARS-CoV-2 infection in household contacts and nonhousehold contacts in study of SARS-CoV-2 variants and age-dependent infection rates over 4 study periods. A–D) Average number of household contacts per index case-patient. E–H) Infection rates for each age group among household contacts. I–J) Average number of nonhousehold contacts per index case-patient. M–P) Infection rates for each age group among nonhousehold contacts.
